# A rare case of Polyarteritis Nodosa associated with autoimmune hepatitis: a case report

**DOI:** 10.1186/s41927-021-00188-1

**Published:** 2021-05-26

**Authors:** Freda Kennedy, Rachel Kapelow, Bilge D. Kalyon, Nitzan C. Roth, Arvind Rishi, Maria-Louise Barilla-LaBarca

**Affiliations:** 1 Department of Medicine, Northwell Health, 300 Community Drive, Manhasset, NY 11030 USA; 2 Division of Rheumatology, Department of Medicine, Northwell Health, 865 Northern Boulevard, Suite 302, Great Neck, NY 11021 USA; 3 Department of Surgery, Northwell Health, 300 Community Drive, Manhasset, NY 11030 USA; 4 Sandra Atlas Bass Center for Liver Diseases and Division of Hepatology, Department of Medicine, Northwell Health, 400 Community Drive, Manhasset, NY 11030 USA; 5 Department of Pathology and Laboratory Medicine, 2200 Northern Boulevard, Suite 104, Greenvale, NY 11548 USA

**Keywords:** Autoimmune hepatitis, Polyarteritis nodosa, Cyclophosphamide, Azathioprine

## Abstract

**Background:**

Polyarteritis nodosa is a type of vasculitis affecting medium- and small-sized arteries that has been associated with hepatitis B but does not have an established relationship with autoimmune hepatitis. Here we report the case of an adult patient with autoimmune hepatitis who, shortly after diagnosis, developed life-threatening polyarteritis nodosa.

**Case presentation:**

A 45-year-old woman was diagnosed with autoimmune hepatitis after initially presenting with a two-month history of fatigue, nausea, and anorexia and a three-week history of scleral icterus. Her liver biopsy showed mild portal fibrosis and her liver chemistries improved with prednisone and azathioprine. Three months later, she presented to the emergency department with fever, bilateral ankle pain, rash, oral ulcers, and poor vision. Physical examination was notable for erythema nodosum, anterior uveitis, retinal vasculitis, and frosted branch angiitis (frosted branch angiitis (a widespread florid translucent perivascular exudate). She subsequently developed repeated episodes of ischemic acute bowel necrosis that required multiple surgeries and extensive small bowel resections. Surgical pathology of the small bowel resection revealed ischemic necrosis, medium and small vessel vasculitis with microvascular thrombi consistent with polyarteritis nodosa. Azathioprine was discontinued and she was treated with pulse steroids followed by a prednisone taper, cyclophosphamide, and intravenous immune globulin with overall improvement in her symptomatology. Since her hospitalization, she has been maintained on low-dose prednisone and mycophenolate mofetil.

**Conclusions:**

In patients with recent diagnosis of autoimmune hepatitis, there should be a modest suspicion for concomitant polyarteritis nodosa if symptoms and signs of multisystem vasculitis develop.

## Background

Polyarteritis nodosa (PAN) is a rare inflammatory vasculitis that affects both men and women equally with a prevalence of 5–10 patients per million. PAN typically involves the medium and small arteries with arterial inflammation leading to vessel narrowing and subsequent formation of aneurysms and microaneurysms. Historically, PAN has been found to be frequently associated with hepatitis B (HBV) infection, occurring at a rate of 30% in that population [1]. However, with the introduction of the HBV vaccination, the incidence of PAN has been greatly reduced [2]. Organs and areas of the body that are characteristically affected by PAN include the kidneys, mesenteric blood vessels, small intestine, peripheral nervous system, and skin with smaller incidences of involvement of the testicles, ovaries, breasts and coronary arteries. Involvement of the liver is very unusual and has a variable presentation ranging from elevated liver enzymes to the development of hepatic aneurysms [3].

Autoimmune hepatitis (AIH) is an inflammatory liver disease characterized histologically by lymphoplasmacytic hepatitis with varying degrees of inflammation and fibrosis. Typical symptoms, if present, include fatigue, myalgias, right upper quadrant discomfort, and sometimes development of jaundice. It often presents indolently but can also cause fulminant liver failure. AIH is more common among women and can be associated with other autoimmune diseases such as inflammatory bowel disease [4]. There is no strong known correlation between PAN and AIH as the simultaneous occurrence of both diseases in an adult patient has been described in only two other reports in the literature [5, 6]. Here we report the unusual case of a non-HBV associated PAN occurring concurrently with AIH.

## Case presentation

**Fig. 1:**
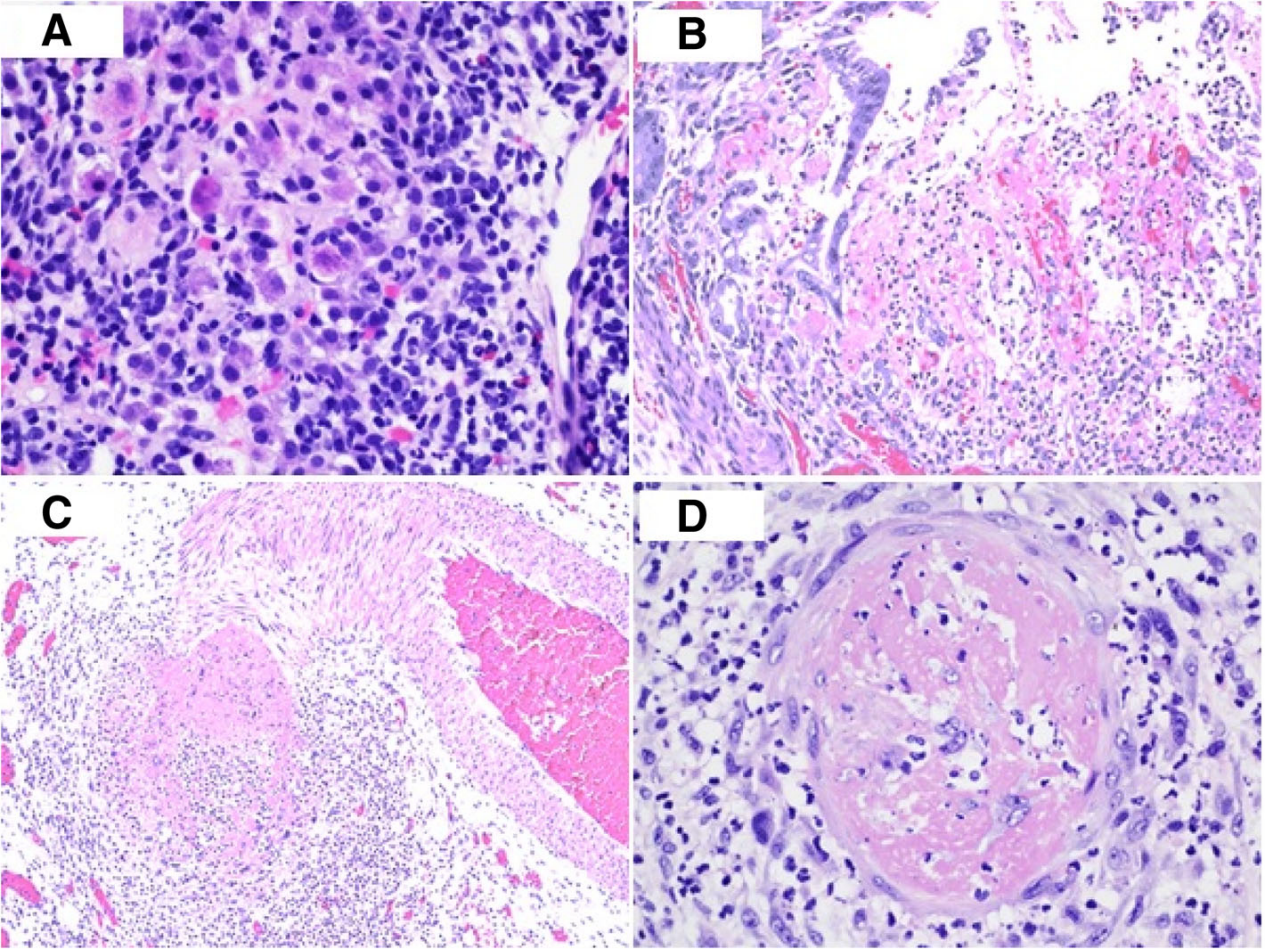
**a** Liver needle biopsy showing marked interface activity with lymphoplasmacytic inflammation and apoptotic hepatocytes (H&E stain × 400 magnification). **b** Small bowel with extensive ischemic necrosis and fibrinopurulent exudate on the mucosal surface (H&E stain × 200 magnification). **c** Medium sized muscular blood vessel with fibrinoid necrosis and neutrophilic inflammation of the vessel wall (H&E stain × 100 magnification). **d** Small capillary with thrombotic microangiopathy and acute inflammation with leukocytoclastic-like inflammatory response (H&E stain × 400 magnification)

A 45-year-old previously healthy woman on no medications presented with a two-month history of fatigue, nausea, anorexia, and constipation and a three-week history of scleral icterus. Liver enzymes were consistent with hepatitis (with peak levels of serum aspartate aminotransferase and alanine aminotransferase of 642 and 549 U/L, respectively) and hyperbilirubinemia to 3.8 mg/dL but with preserved liver synthetic function. Alkaline phosphatase levels were elevated on initial testing and remained elevated throughout the disease course. Abdominal sonogram showed a normal appearance of the liver, spleen, gallbladder, and biliary tree. Laboratory work-up was notable for a positive antinuclear antibody (ANA) with 1:640 titer and speckled pattern, a positive anti-smooth muscle antibody (ASMA) with 1:80 titer, and a markedly elevated immunoglobulin G of 3268 mg/dL, suspicious for AIH. There was no evidence of acute or chronic viral hepatitis. Hepatitis E was negative. She underwent percutaneous liver biopsy that revealed moderate to severely active hepatitis, necroinflammatory bridging necrosis with interface activity and moderate to marked portal inflammation comprising numerous plasma cells and few lymphocytes. Apoptotic hepatocytes were identified (Fig. 1A). There was minimum F1 portal fibrosis. The histological pattern of inflammation, autoantibodies and clinical presentation were consistent with AIH. The patient began treatment with prednisone and azathioprine 1.2 mg/kg daily since her AIH diagnosis in 4/2019, with rapid improvement in her alkaline phosphatase and transaminase levels. She was then discontinued on azathioprine in 7/2019 when diagnosed with PAN. At that time, she received pulse steroids, IVIG 0.4 mg/kg daily × 5 days, and CYC August to November 2019. She continued on a steroid taper for several months.

She was doing well until 3 months later when she acutely developed fever, bilateral ankle pain, rash, oral ulcers, and poor vision. She denied dyspnea, chest pain, irregular heartbeats, lymphadenopathy, weight loss, rash, genital ulcers or eye pain. Examination was notable for bilateral erythema nodosum on her legs, anterior uveitis, retinal vasculitis, and frosted branch angiitis. Her prednisone, which previously had been tapered as her liver function tests improved, was increased and rheumatologic workup was initiated and subsequently returned negative for lupus, sarcoid and Behcet’s (Table 1). During this workup, she developed new onset of uncontrolled nausea and emesis associated with severe diffuse abdominal pain with peritoneal signs on examination. She was found to have proximal small bowel obstruction involving the jejunum with thickened jejunal loops and developing ascites on CT imaging. Emergent exploratory laparotomy resulted in resection of 25 cm of patchy to full-thickness necrotic bowel. Histological examination of the resected segment of small bowel showed extensive areas of necrosis (Fig. 1B), vascular congestion, fibrinoid necrosis of small to medium sized blood vessels (Fig. 1C) and foci of small sized blood vessels and capillaries with fibrinous thrombi, acute neutrophilic inflammation and leukocytoclastic-like nuclear debris of the inflammatory cells (Fig. 1D), morphologically consistent with thrombotic fibrinoid vasculitis consistent with PAN. No IgG4 staining was performed in this case as the pattern of inflammation and fibrosis were not of IgG4 disease.

**Table 1:** Summary of Labs and Serologic Markers

CBC	within normal limits
BMP	within normal limits
PT/PTT/INR	within normal limits
dsDNA	negative
Sm/RNP	negative
Anti-SS-A, Anti-SS-B	negative
ANCA	negative
MPO	negative
PR-3	negative
ACE	negative
(HLA)-B51	negative
(HLA)-B52	negative
CCP	negative
Cryoglobulins	negative
C3, C4	within normal limits
RF	19 U/mL
ESR	105 mm/hr
CPK	within normal limits
HIV	negative
CMV	negative
EBV	negative
Parvovirus B19	negative
Lyme	negative
Urinalysis	Bland, no proteinuria

Her prolonged 6-month hospital course was complicated by recurrent small bowel ischemia and necrosis requiring additional resections, duodenal stump blowout with enterocutaneous fistula formation, and multiple infections. Treatment with pulse steroids and cyclophosphamide was begun, but was held after the second dose due to bacteremia. Adjuvant immunomodulatory therapy with intravenous immune globulin (IVIg, 0.4 g/kg/day for 5 days) was added until she was able to resume cyclophosphamide. She received a total of four doses of cyclophosphamide. She experienced a fair clinical response that allowed for discharge from the hospital still on total parenteral nutrition. Enteral nutrition was gradually re-introduced successfully as an outpatient and she was transitioned to mycophenolate mofetil (MMF) for maintenance therapy. Steroids were gradually weaned off. Notably, she achieved biochemical remission of her AIH while on that maintenance regimen, though with persistent mild (less than two times the upper limit of normal) elevations of serum alkaline phosphatase. Her duodenocutaneous fistula closed spontaneously, and she now tolerates a regular diet. One year later, she is free of gastrointestinal symptoms or skin manifestations and has not had progression of her ocular pathology, though she has residual visual loss.

## Discussion and conclusions

PAN is a medium- and small-artery vasculitis, which is typically associated with HBV infection, hepatitis C virus infection, HIV, and hairy cell leukemia. Although the overall incidence of PAN is decreasing worldwide, likely due to successful HBV vaccination strategies [2], it is now being recognized with more regularity in non-HBV-infected patients. To our knowledge, this patient represents only the third case of PAN in association with AIH in an adult patient [5, 6]. In our patient, PAN developed soon after the development of AIH, consistent with prior reports, and therefore suggesting a possible pathogenetic link. Our patient’s ophthalmic and dermatologic symptoms were atypical for either AIH or PAN (Table 2). Although ocular involvement can occur in approximately 20% of PAN cases, it is usually scleritis [7] as opposed to our patient who had uveitis and frosted branch angiitis, a rare form of retinal vasculitis. Furthermore, she displayed erythema nodosum which is an atypical skin finding in PAN [8]. Common skin findings in PAN include livedo and leukocytoclastic vasculitis, which our patient did not have. Finally, all her symptoms were in the context of treatment for AIH with azathioprine. Workup and consideration of other autoimmune inflammatory disease more often associated with uveitis and erythema nodosum such as sarcoid and Behcet’s was negative, and diagnosis was largely realized on histological examination. It should be noted that a celiac axis angiogram was not performed and is a limitation in this report.

**Table 2:** Signs and Symptoms of PAN and AIH

Organ systems	Polyarteritis Nodosa	Autoimmune Hepatitis
Ophthalmic	Scleritis	Jaundice
Cardiac	Pericarditis, myocarditis, myocardial infarction	None
Gastrointestinal	Intestinal necrosis and perforation	Hepatomegaly, abdominal discomfort, nausea, vomiting, diarrhea
Renal	Hypertension	Edema
Skin	Rashes, swelling, necrotic ulcers, and subcutaneous nodules	Jaundice, rash
Neurologic	Strokes, seizures	Sensory neuropathy
Constitutional	Fever, fatigue, weakness, loss of appetite, unintentional weight loss, myalgias, arthralgias	Fatigue, myalgias, arthralgias

We present an interesting and unusual co-existence of two rare diseases, AIH and PAN, in an adult patient. Treatment of a patient with the concomitant diagnoses of PAN and autoimmune hepatitis can be very challenging. Prognosis of PAN that is untreated remains poor; however, with treatment it has an 80% five-year survival rate [9]. Commonly, the treatment of PAN involves an aggressive regimen that includes high-dose prednisone and cyclophosphamide; while the treatment of autoimmune hepatitis includes the use of glucocorticoids and azathioprine for at least 24 months. Both diseases are treated similarly with glucocorticoids in conjunction with an immunosuppressive agent. To note, this patient was not tested for deficiency of adenosine deaminase 2 (DADA2). Many cases of severe non-HBV PAN have been found to be secondary to DADA2 [10]; additionally, DADA2 has also been described to cause hepatitis and intestinal necrosis [11], like the one reported in this case. In patients with recent diagnosis of autoimmune hepatitis, there should be a possible suspicion for concomitant polyarteritis nodosa if symptoms and signs of multisystem vasculitis develop; however, it should be noted that these observations could all be co-incidental. In this particular patient, successful remission was achieved with the use of both immunosuppressive agents and steroids during her hospitalization. Currently, she is on MMF 2 g daily and off steroids. She is reportedly doing well.

## Data Availability

Data sharing is not applicable to this article, because no datasets were generated or analyzed during the present study.

## Abbreviations

PAN: Polyarteritis nodosa
AIH: Autoimmune hepatitis
ANA: Antinuclear antibody
AMA: Antismooth-muscle antibody
RF: Rheumatoid factor
ESR: Erythrocyte sedimentation rate
CRP: C-reactive protein
